# Tracing coalition changes in knowledge in and engagement with childhood obesity prevention to improve intervention implementation

**DOI:** 10.1186/s12889-022-14208-3

**Published:** 2022-09-30

**Authors:** Travis R. Moore, Mark C. Pachucki, Erin Hennessy, Christina D. Economos

**Affiliations:** 1 Friedman School of Nutrition Science and Policy, ChildObesity180, Tufts University, 150 Harrison Ave, Boston, MA 02111 USA; 2 Department of Sociology, Computational Social Science Institute, University of Massachusetts Amherst, Amherst, MA 01003 USA

**Keywords:** Childhood obesity, Prevention, RSiena, Simulation, Community coalition, Implementation, Social network

## Abstract

**Background:**

While most coalition research focuses on studying the effects of peer relationship structure, this study examines the coevolution of coalition structure and behavior across three communities in the U.S. with the goal of identifying coalition dynamics that impact a childhood obesity prevention intervention.

**Methods:**

Over two years (2018–2020), three communities within the U.S. participated in a childhood obesity prevention intervention at different times. This intervention was guided by the Stakeholder-Driven Community Diffusion theory, which describes an empirically testable mechanism for promoting community change. Measures are part of the Stakeholder-driven Community Diffusion (SDCD) survey with demonstrated reliability, which include knowledge of and engagement with childhood obesity prevention and social networks. Data from three coalition-committees and their respective networks were used to build three different stochastic actor-oriented models. These models were used to examine the coevolution of coalition structure with coalition behavior (defined a priori as knowledge of and engagement with obesity prevention) among coalition-committee members and their nominated alters (Network A) and coalition-committee members only (Network B).

**Results:**

Overall, coalitions decrease in size and their structure becomes less dense over time. Both Network A and B show a consistent preference to form and sustain ties with those who have more ties. In Network B, there was a trend for those who have higher knowledge scores to increase their number of ties over time. The same trend appeared in Network A but varied based on their peers’ knowledge in and engagement with childhood obesity prevention. Across models, engagement with childhood obesity prevention research was not a significant driver of changes in either coalition network structure or knowledge.

**Conclusions:**

The trends in coalition Network A and B’s coevolution models may point to context-specific features (e.g., ties among stakeholders) that can be leveraged for better intervention implementation. To that end, examining tie density, average path length, network diameter, and the dynamics of each behavior outcome (i.e., knowledge in and engagement with childhood obesity prevention) may help tailor whole-of-community interventions. Future research should attend to additional behavioral variables (e.g., group efficacy) that can capture other aspects of coalition development and that influence implementation, and to testing the efficacy of network interventions after trends have been identified.

## Contributions to the literature

- Research has shown that community coalitions are vital to whole-of-community interventions. Most coalition research focuses on studying the effects of peer relationship structure on intervention success. However, we found that studying the coevolution of coalition structure *and* behavior using stochastic actor-oriented models paints a fuller picture of what might drive knowledge in and engagement with childhood obesity prevention.
- We found that social network structure more often drove changes in coalition-committee member and community member knowledge in childhood obesity prevention than the behavior variables themselves. We found considerable variability in the behavior variables in each model across communities largely based on differences in first-degree alter behaviors.
- These findings contribute to recognized gaps in the literature, including ascertaining the potential drivers of coalition knowledge of and engagement with childhood obesity prevention.

## Background

Complex health issues such as childhood obesity require tailored, systems-oriented action [1, 2]. Whole-of-community (WoC) childhood obesity prevention interventions hold promise by synergistically targeting multiple weight-related behaviors at multiple levels of influence (e.g., families, community-based organizations, and local government) [3, 4]. These whole-of-community interventions offer stakeholders from varying levels of service and settings opportunities to work collectively toward improving child health [4]. Their promise has been documented [4–6], and researchers are working to improve their implementation by studying the factors that drive their success.

Community coalitions (hereunder referred to as coalitions), partnerships that include stakeholders from organizations that represent multiple sectors (e.g., public health, schools, community-based organizations), can be essential to implementing WoC interventions [7, 8]. Coalitions bridge traditionally siloed stakeholders and organizations to (a) generate broad and diverse community representation and (b) increase stakeholder capacity to implement a portfolio of evidence-based strategies to effect mid- and down-stream changes [9, 10]. In childhood obesity prevention, coalitions have held implementation leadership and coordination roles in several childhood obesity prevention trials, contributing to local capacity building and intervention sustainability [3, 11, 12]. Thus, community coalitions are often well positioned intermediaries that organize and mobilize stakeholders to act on preventing childhood obesity by supporting cross-sector collaboration and research-to-practice translation [8, 10, 13].

To maximize coalitions’ unique position in communities and role in sustaining WoC interventions, some researchers are examining stakeholders’ knowledge, engagement, and social networks. Knowledge is conceptualized as stakeholders’ understanding of community-wide efforts to prevent childhood obesity and refers to several conceptual domains including the problem of childhood obesity, the modifiable determinants of childhood obesity, stakeholders’ roles in childhood obesity prevention interventions and knowledge of multi-setting components, how to intervene to achieve sustainability, and available resources to address the issue [14]. Engagement is conceptualized as stakeholders’ enthusiasm and agency for preventing childhood obesity in their community and refers to five conceptual domains including exchange of skills and understanding, willingness to compromise and adapt, ability or capacity to influence the course of events and others’ thinking and behavior, action of directing and being responsible for a group of people or course of events, and the belief and confidence in others [14]. Stakeholders’ social networks are simply their relationships with other stakeholders. In this study, their relationships are defined by whether they discuss childhood obesity prevention with one another. Measured by social network surveys such as the Stakeholder-driven Community Diffusion Survey [14], stakeholders’ social networks have potential shape the creation of new collaborative activities, knowledge and engagement exchange, transmission of local information and advocacy, and access to resources distributed throughout the coalition [15, 16]. Taken together, engagement with childhood obesity prevention motivates stakeholders to share their knowledge with others in their social network and represents stakeholders’ desires and ability to translate their knowledge into effective action for WoC interventions.

Network science allows researchers to examine relationships between stakeholders’ knowledge, engagement, and social networks by categorizing these characteristics into coalition structure and coalition behavior. Coalition structure is defined here as the various types and conformations of relationships among stakeholders. A coalition’s structure is characterized by network metrics such as degree (the number of connections a stakeholder has), betweenness (which detects the amount of influence a stakeholder has over the flow of information in a network; generally, the higher the betweenness, the more a stakeholder acts as a key bridge of information between other stakeholder within the network), or closeness (the distance each stakeholder is from all other coalition members in the network). A coalition’s behavior is defined here using the broad characterization found within the network simulation literature, which states that the term “behavior” should not be taken literally. Thus, coalition behavior is defined as any coalition attribute other than their structural attributes. This means that behavior can be understood in conventional terms (e.g., smoking) and in the context of simulation (e.g., changes in perceptions or attitudes). In this paper, we define behavior as “knowledge” and “engagement”, which represent stakeholders’ knowledge in and engagement with childhood obesity prevention, attributes that coevolve alongside changes in their structural relationships with one another.

Most of the research on coalition formation over time has focused on structure. Many such studies have been limited to retrospective and cross-sectional designs but have made meaningful strides in exploring coalition structures using social network analysis [17, 18]. For instance, some researchers have suggested that less hierarchical coalitions (lower network centralization) are able to build members’ trust and agency [10, 19, 20]. Additionally, in a study of substance abuse prevention coalitions, networks with more connections among members (greater network density) had lower rates of adopting evidence-based programs—perhaps due to challenges in accessing and mobilizing innovative thinking and resources external to the coalition that would benefit implementation [21, 22]. This finding is counterintuitive to conventional wisdom that suggests greater density may positively influence diffusion because there are many paths between those who are connected; if a network becomes sparser (i.e., less dense), then diffusion between a large number of people can become more difficult.

As documented by both Bess and Korn, coalition network structures evolve throughout prevention interventions [10, 23]. Researchers who prospectively and longitudinally examine the structural changes among stakeholder ties in coalitions try to identify the conformation that leads to improved prevention intervention planning, implementation, and sustainability [23]. Using exponential random graph models, Korn and colleagues found that a coalition participating in a childhood obesity prevention intervention experienced changes to its network structure. For example, stakeholder networks within the coalition had the most connections and a high level of control over information at the beginning of the intervention. In another example, by the end of the intervention stakeholder, ties were increasingly perceived as influential and siloed (i.e., connections between stakeholders did not span other stakeholder groups or organizations). These results indicate that coalition structure evolves, highlighting the need for additional longitudinal research that (1) incorporates and closely monitors structural variables as well as coalition behavior; and (2) focuses on variables related to childhood obesity prevention.

Social network simulation is a newer development, with researchers using simulations to expand coalition network research beyond their structural components to include coalition behavior, and ultimately model complex interactions between coalition structure and behavior [24] [16]. For instance, in Kasman and colleagues’ research, an agent-based model was developed to retrospectively simulate the diffusion of knowledge about and engagement with obesity prevention efforts through the community [16]. By including social network model inputs such as group membership and group connectivity, the model was able to provide outputs that met the evaluation criteria of increasing simulated knowledge and engagement, a form of stakeholder behavior. More research is needed to identify the salient coalition peer-relationships involved in diffusion behavior that builds from existing literature on how coalitions evolve over time.

Thus, building on prior theory and research on coalition network structure and behavior, this study examines changes in a range of network structure attributes in relation to knowledge in and engagement with childhood obesity prevention. Over time, we expected to observe changes in coalition structure and behavior unique to each community that could point to (1) how coalition formation changes over time in general; and (2) how an intervention could be tailored to improve the adoption of childhood obesity prevention research within coalitions more specifically. The aim of this study is to deepen understanding of the associations between changes in coalition peer relationships and changes in their knowledge in and engagement with childhood obesity prevention. This study provides the first empirical prospective examination of cross-coalition network structure and behavior during the design and implementation of a WoC intervention to prevent childhood obesity.

## Methods

### Catalyzing communities

We analyzed social network data collected over two years (from 2018 to 2020) from three communities involved in Catalyzing Communities: an ongoing WoC childhood obesity prevention intervention [8, 25]. Table 1 describes each included coalition-committee and each coalition-committee’s community characteristics. Reporting follows the STROBE checklist.

**Table 1:** Summary of community and coalition-committee characteristics

Community	1	2	3
**Community characteristics (2019)**			
Population estimate	514,213	46,655	385,282
Land area (mi ^2^)	785.0	4.8	82.5
Median household income (USD)	$53,739	$48,704	$20,407
Foreign born (%)	7.9	50.4	5.9
**Race and ethnicity (%)**^a^			
Hispanic or Latino (all races)	8.8	57.4	11.9
NH White	69.0	32.6	40.0
NH Black or African American	18.0	2.6	48.8
NH American Indian and Alaska Native	0.2	0.0	0.5
NH Asian	2.2	3.8	2.6
NH Native Hawaiian and Other Pacific Islander	0.1	0.1	0.1
NH some other race	0.1	0.2	0.1
NH two or more races	1.7	3.4	1.8
**Baseline Coalition-Committee characteristics**			
Coalition-committee size (n)	18	15	12
Density	0.08	0.27	0.14
Transitivity	0.45	0.37	0.14
Number of ties	27	28	11
Mean(SD) degree	1.42(1.89)	3.73(1.12)	1.69 (0.98)
Network diameter	4	3	3
Average path length	2.33	1.81	1.94
Bachelor’s degree and above (%)	50.0	50.0	18.0
Female (%)	84.0	78.0	96.0
Target child age of intervention	0–18 y	0–18 y	0–8 y
Coalition Focus Area(s) ^1^	Policy, practice, and environmental change; Health equity; WIC ^2^ participation; human-centered messaging	Increase utilization of community resources among underserved populations; increasing youth physical activity; mental health	Advocacy, communications, evaluation of early care programs

^a^From the American Community Survey [26]

The Catalyzing Communities project design and methods are described elsewhere [25, 27]. In brief, Catalyzing Communities investigates the attributes and processes of newly formed coalitions (the “Coalition-Committee”) that are convened for the study and comprised of stakeholders from an array of sectors (e.g., community-based organizations, community members, hospitals, schools, philanthropy) serving children and their families. It is based on the Stakeholder-Driven Community Diffusion Theory, which describes an empirically testable mechanism for promoting community change whereby knowledge of and engagement with childhood obesity within a group of convened stakeholders can diffuse through their social networks and lead to changes in policies, systems, and environments that have been shown impact children’s behaviors and health outcomes [28, 29]. Our SDCD-informed intervention employs group model building and technical assistance with convened stakeholders to build knowledge, engagement, and the use of research evidence in community-led actions [27]. Our initial studies have been shown to increase knowledge of and engagement with childhood obesity prevention efforts among stakeholders [14]. While this study uses a retrospective approach to understand patterns in coalition structure and behavior using stochastic actor-oriented models, we have also prospectively examined diffusion using agent-based modeling [14].

Reported previously, we identified changemakers, those who work closely with an array of stakeholders on childhood obesity prevention, through past partnerships and prior research collaborations [8]. Two to three changemakers per community were selected based on their capacity to participate in the SDCD-informed intervention, their community characteristics (race, ethnicity, median household income, population, and land area), and perceived readiness to participate. We worked closely with changemakers in each community to identify the stakeholders who should be in each coalition-committee. Changemakers identified between 12 and 18 stakeholders across communities to participate in the intervention, described elsewhere [25]. These stakeholders formed the coalition-committee that participated in the intervention. Selection of stakeholders was guided by their capacity and readiness to participate as well as their representative sector.

**Fig. 1:**
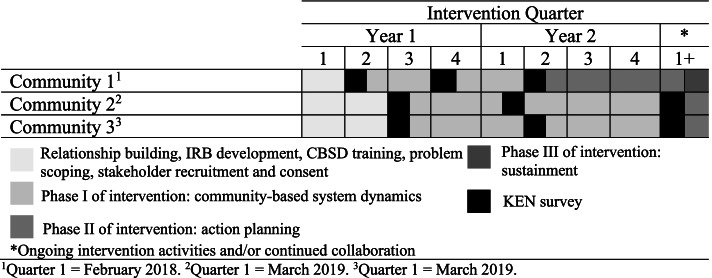
Timeline of the SDCD theory-informed intervention across three communities

Figure 1 includes a timeline and description of the Catalyzing Communities project with three communities and corresponding measurement waves (W1-W3). This table is provided to contextualize the results of the simulation.

#### Data collection: Sampling and participants

**Fig. 2:**
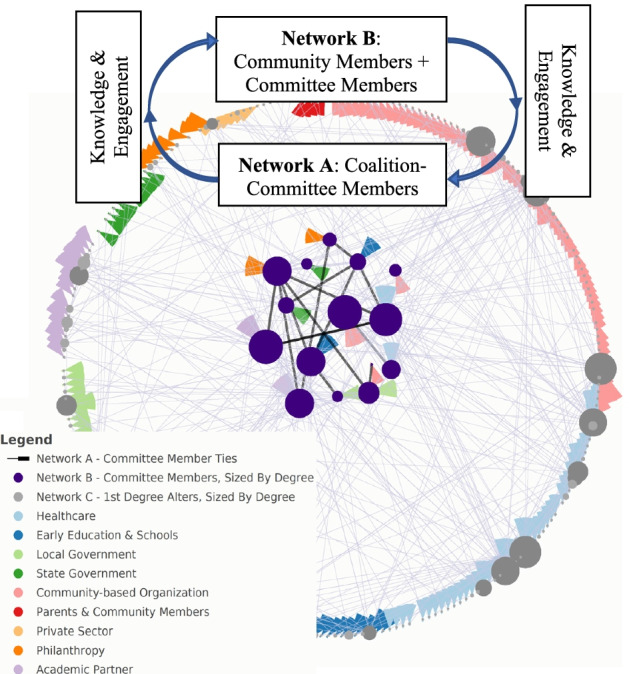
Conceptual model of one community participating in the stakeholder-driven community diffusion theory-informed intervention *Note*. This conceptual model is based on real data from the Catalyzing Communities project. Our analyses differentiate between Network A, which consists of coalition-committee members who directly participate in the SDCD theory informed intervention (purple nodes), and Network B, which consists of coalition-committee members and their first-degree alters. Each node is colored by its corresponding sector affiliation. Together, these networks create the intervention system wherein knowledge in and engagement with childhood obesity prevention is hypothesized to diffuse from Network A to Network B in cycles over time. Adapted from Moore et al., 2021 [8]

We employed a snowball sampling approach initiating from coalition-committee members with the goal to observe community-wide connections related to early childhood obesity prevention. The network included coalition-committee members and nominees of coalition-committee members (“first-degree alters”), collectively named “stakeholders” when describing all network members (Fig. 2). We refer to ties among coalition-committee members only as Network B in our analyses. We refer to ties among coalition-committee members and their first-degree alters as Network A in our analyses. Network A and B were developed for each community coalition. This demarcation is grounded in how the SDCD theory conceptualizes the process of diffusion, from coalition-committee member (Network B) to broader community members (Network A).

Coalition-committee members completed three web-based surveys described below, nominating up to 20 individuals each survey (“defined as first degree alter community members”). As seen in Fig. 1, the first survey was administered after the recruitment/relationship building phase and prior to Phase I of the intervention. Phase 1 consists of community-based system dynamics where participants are convened in a series of group model building sessions to identify an issue of local concern, construct diagrams of the drivers of that issue, and begin thinking about planning and addressing that issue in Phase 2. The second survey was administered halfway through Phase I of the intervention. The third survey was administered directly after Phase I of the intervention or in the beginning of Phase 2. First-degree alter surveys were administered to nominees of coalition-committee members at the same timepoints.

Survey respondents (including both coalition-committee members and their nominees) reported nominees’ organization or department and title. Respondents did not report nominees’ contact information or individual characteristics (e.g., gender) to minimize respondent burden and potential discomfort of reporting nominees’ personal information. Using the data provided, successful recruitment of first-degree alters was contingent on the research team’s ability to obtain accurate email addresses through online searches and existing contacts (e.g., changemakers). This approach captured contact details for approximately 80% of first-degree alters at each round. As with most social network recruitment, recruitment was inconsistent for first-degree alters across time points depending on whether coalition-committee members named the same and same number of first-degree alters at each time point. However, this variability was not significant enough to destabilize the simulations included in this study, as indicated by the Jaccard Indices (JI) in Tables 4 and 5, which need to be above 0.25 [30].

The Tufts University Institutional Review Board approved all study procedures in each community. Coalition-committee members provided written informed consent electronically and received a stipend. First degree alters provided informed consent electronically and were offered a gift card per survey.

#### Measures

Knowledge, engagement, and social networks were assessed via the Stakeholder-driven Community Diffusion survey with demonstrated reliability [14].

##### *Knowledge*

Survey respondents were asked to score their understanding of childhood obesity prevention in their community (broadly termed “knowledge”). Knowledge of the topic of childhood obesity prevention is assessed on a 5-point Likert scale from “strongly agree” to “strongly disagree” (internal scale consistency Cronbach’s alpha = 0.86) across five domains: (1) knowledge of the problem (5 questions); (2) modifiable determinants of the problem (5 questions); (3) stakeholders’ roles related to addressing the problem in their community (3 questions); (4) sustainable intervention approaches (7 questions); and, (5) knowledge of available resources (4 questions).

##### *Engagement*

Survey respondents were asked to score their level of enthusiasm for and commitment to childhood obesity prevention (broadly termed “engagement”). Engagement is conceptualized as enthusiasm and agency for the topic of childhood obesity prevention (internal scale consistency Cronbach’s alpha = 0.92). Engagement comprises five domains: (1) exchange dialogue and mutual learning (6 questions); (2) flexibility (4 questions); (3) influence and power (4 questions); (4) leadership and stewardship (10 questions); (5) trust (4 questions).

##### *Network structure*

Survey respondents were asked to provide the names of up to 20 people with whom they discuss issues related to childhood obesity prevention. These nominations are the foundation of the overall network structure. For surveys 2 and 3, coalition-committee members were prompted with a list of nominees from their prior survey responses and were instructed to renominate any current ties.

#### Data preparation and analysis

Each network was treated as a non-directed network, under a reasonable assumption that if one stakeholder nominated another stakeholder, then they were mutual friends and considered a tie in the analyses. Conceptually, this means that if one stakeholder nominated another stakeholder, they were considered two stakeholders who discuss childhood obesity prevention with each other. All ties and stakeholders in the analyses were retained and represented as our best approximation of each community’s network structure related to childhood obesity prevention. Ties emerged from three scenarios: (1) stakeholders A and B both responded to the survey and nominated each other (counts as one tie); (2) A and B responded, but A nominated B or B nominated A; (3) only A or B responded and nominated the other. Structural zeroes were imputed where there was no tie from stakeholder to stakeholder to handle stakeholders joining or leaving the network between the start and the end of observation.

Longitudinal analyses of change related to network structure and behavior of coalition-committees only (Network B) and coalition-committees and their first-degree alters (Network A) were conducted in RSiena (Simulation investigation for Empirical Network Analysis, [30]). RSiena can model network coevolution by employing stochastic actor-oriented models, a type of agent-based model oriented specifically to uncover the reciprocal influence of network structure and behavior, to estimate parameters of network dynamics using longitudinal network data. These parameters operate in linear combination to predict network coevolution. This type of model is positioned to evaluate how actor networks and actor traits may simultaneously change. Although ‘engagement’ is more strictly behavioral than ‘self-report of knowledge’, both traits are amenable to analyzing how their change may be co-occurring with network change.

After the data for each community were prepared, effects were selected based on standard requirements for SIENA models as well as theoretical considerations. Following the recommendation of Snijders et al. (2010), a forward selection approach was used for model specification. The forward selection approach optimizes good estimates by iteratively adding effects to the model, dropping effects if they are insignificant. RSiena non-directed models require the inclusion of degree and, as standard practice, includes at least one transitivity effect. In addition to these required structural effects, we included transitive triplets (i.e., a common relationship structure between three individuals) and degree assortativity (i.e., preference for a stakeholder to connect to others that are similar in degree) to test for local hierarchy. Final models for each community were selected based on the inclusion of significant effects, dropping other effects to obtain maximally explanatory models with greatest model parsimony. Thus, each model contains only significant effects, as adding other effects adjusts the estimates of the other parameters. Finally, each model is based on an important assumption^1^ about tie formation that we selected based on our closest approximation about how stakeholder’s and their alters form or discontinue ties over time.

Beyond model convergence tests, goodness of fit tests were run using the function “sienaGOF” [31], that enables testing the fit of RSiena models with respect to auxiliary statistics of networks. These auxiliary statistics, such as geodesic distribution, triad census, and indegree distribution, are not explicitly fit by a particular model effect, but they are important features of the network to represent by the probability model [32]^2^. All model effects were above the customary *p* = 0.05 threshold, indicating acceptable model fit.

## Results

Three different coalition-committees who represent a subgroup within their larger community coalition participated in the intervention. Demographics-including race, ethnicity, gender, mean age, years of experience, and sector-of each coalition-committee, which remains the same at each time point, is reported in Table 2. Each coalition-committee was asked to nominate those with whom they discuss childhood obesity prevention at three time points; thus, the sample for coalition-committees does not change across time points while their nominations, representing their larger professional network, does change across time points. Demographics for this larger professional network are not reported due to missing data. Variability exists across each coalition-committee demographics, with Community 1 and 3 containing a greater percentage of Black or African Americans; Community 2 containing a greater percentage of individuals who identify as Hispanic; and Community 2 consisting entirely of individuals from the community-based organization sector.

**Table 2:** Coalition-committee demographics across communities

	**Coalition-Committees**		
	**Community 1 (*n* = 18)**	**Community 2 (*n* = 15)**	**Community 3 (*n* = 12)**
Race (%)			
Asian	0	8.3	7.69
Black or African American	21.05	8.3	38.46
White	78.95	75	53.85
Other	0	0	0
Ethnicity (%)			
Hispanic	5.26	16.67	0
Gender (%)			
Female	78.95	66.67	91.67
Male	21.05	33.33	8.33
Other	0	0	0
Age (mean)	49	44	49
Years of experience (mean)	17	19	20
Sector (%)			
Community-based Organization	15.79	100	15.38
Early Education and Schools	10.53	0	46.15
Healthcare	21.05	0	7.69
Local Government	5.26	0	7.69
Philanthropy	10.53	0	7.69
Private Sector	0	0	7.69
State Government	26.32	0	7.69
Academic Partners	0	0	0

### Network A

Descriptively, we observe that Community 1–3 Network A (coalition-committee egos + alters) density decreases, average path length increases, and network diameter increases, indicating that the network is becoming sparser over time (Table 3).

**Table 3:** Characteristics of network A and network B across communities

	Community 1			Community 2			Community 3		
	R1	R2	R3	R1	R2	R3	R1	R2	R3
Network A									
Degree (SD)	1.42 (1.89)	1.21 (0.89)	1.11 (0.98)	3.73 (1.12)	2.73 (1.06)	2.29 (1.22)	1.69 (0.98)	2.00 (1.27)	1.59 (1.55)
Density	0.079	0.067	0.061	0.267	0.124	0.122	0.141	0.167	0.122
Transitivity	0.44	0.24	0.27	0.37	0.32	0.46	0.14	0.29	0.32
Number of ties	27	23	21	28	13	24	11	13	10
Average path length	2.32	2.05	2.14	1.81	2.39	2.97	1.94	2.23	2.20
Diameter	4	4	4	3	5	3	3	5	5
Network B									
Degree (SD)	0.42 (0.02)	0.38 (0.03)	0.32 (0.22)	0.99 (0.98)	0.67 (0.75)	0.68 (0.55)	0.31 (0.21)	0.29 (0.19)	0.29 (0.23)
Density	0.002	0.001	0.001	0.004	0.003	0.003	0.001	0.001	0.000
Transitivity	0.09	0.07	0.05	0.07	0.02	0.03	0.02	0.03	0.04
Number of ties	158	145	122	117	79	80	115	108	105
Average path length	3.48	3.48	3.69	3.24	3.67	3.62	3.52	3.49	3.52
Diameter	6	6	7	5	7	8	6	6	7

Note. Network A refers to coalition-committee members and their 1^st^ degree alters. Network B refers to coalition-committee members only

In estimating co-evolution of network structure with knowledge, we included effects that measured different attributes of egos (egoX^3^) and alters (altX) on network tie formation, and the effect of ego’s engagement with childhood obesity prevention (engagement) on their knowledge in childhood obesity prevention (knowledge; using effFrom). After adding and dropping effects, the final model contained four to five effects for each community. For Community 1–3 Network A structure using knowledge as the dependent behavior variable, fewer connections are made between community members, with a preference for connecting to others who have similar number of ties (Table 4). Results for Network A for Community 1 indicate that actors who are higher on knowledge of childhood obesity prevention become less popular (have fewer ties from alters) over time. However, results for Network A from Community 2 and 3 indicate that actors who are higher on knowledge of childhood obesity prevention form more ties over time. Distinct from other community Network A models, Community 3 Network A includes a significant covariate effect (effFrom) of engagement on knowledge scores over time. This is the main covariate effect and suggests that egos who are more engaged with childhood obesity prevention may experience the most knowledge gain.

**Table 4:** Cross-community summary of coevolution models for knowledge of childhood obesity prevention

	**Community 1 (JI = .382,.267)**^**a**^			**Community 2(JI = .274,.254)**^**a**^			**Community 3(JI = .327,.590)**^**a**^		
	Estimate (SE)	*t*-Ratios	Convergence^b^	Estimate (SE)	*t*-Ratios	Convergence^b^	Estimate (SE)	*t*-Ratios	Convergence^b^
**Network A**									
*Network function of model*									
**Rate constant**									
Period 1	0.984(0.744)			1.999(1.337)			0.564(0.221)		
Period 2	0.722(0.430)			0.956(0.731)			1.035(0.381)		
**Effects**									
Degree	-7.67(0.45)	17.04**	-0.01	-8.71(0.75)	11.61**	0.01	-9.38(0.43)	21.81**	0.03
Transitive Triads	-2.86(0.66)	4.33**	-0.02	-1.86(.81)	2.29**	0.02	-1.39(0.74)	1.88*	0.05
Degree Assortativity	0.87(0.08)	10.88**	-0.01	1.21(0.16)	7.56**	0.00	1.20(0.14)	8.57**	0.01
Effect on Net of Knowledge Alter (altX)	-1.27(0.47)	2.70**	0.01						
Effect on Net of Knowledge Ego (egoX)				1.76(0.45)	3.82**	0.02	1.21(0.09)	13.44**	0.04
*Behavior function of model*									
Linear shape	1.045(0.929)			1.145(0.929)			-0.061(0.329)		
Quadratic shape	-1.621(1.187)			-1.988(1.187)			-1.889(0.437)		
**Effects**									
Engagement on Knowledge (effFrom)							1.28(.65)	1.97*	0.02
**Network B**	**Community 1 (JI = .380,.297)**			**Community 2 (JI = .299,.297)**			**Community 3(JI = .500,.600)**		
*Network function of model*									
**Rate constant**									
Period 1	3.064(1.981)			2.147(1.493)			1.492(1.056)		
Period 2	0.437(0.468)			17.237(16.569)			0.784(0.525)		
**Effects**									
Density	-0.949(0.322)	2.95**	0.07	-2.53(0.768)	3.34**	0.03	-2.87(1.07)	2.68**	0.05
Degree Assortativity	0.28(0.09)	3.11**	0.07	0.34(0.13)	2.62**	0.04	0.55(0.24)	2.29**	0.01
Effect on Net of Knowledge Ego + Alt (egoPlusAltX)				0.98(0.49)	2.00*	0.09	-1.05(0.42)	2.50**	0.06
*Behavior function of model*									
Linear shape	1.202(0.825)			0.735(0.684)			1.602(0.935)		
Quadratic shape	-1.832(0.764)			-1.061(0.923)			-1.034(0.129)		

Note. Blank cells indicate effects dropped to obtain significant values for included effects

^a^Jaccard index for Time 1Time 2 and Time 2Time 3

^b^Excellent model convergence is indicated by convergence t-ratios < .1 (Ripley et al., 2014)

^*^p < .05; **p < .01

For models estimating the co-evolution of network structure with engagement, Community 1 Network A, significant effects were similar to knowledge models, departing slightly with the addition of a significant effect on knowledge from engagement within the behavior function of the model (Table 5). For Community 2 and 3 Network A, the effect of ego engagement scores over time on the network was significantly positive; actors with higher engagement scores tended to form new ties. Unlike Community 1, Community 2 and 3 Network A models for engagement did not include significant covariate effects on the network or behavior functions.

**Table 5:** Cross-community summary of coevolution models for engagement in childhood obesity prevention

	**Community 1 (JI = .383,.267)**^**a**^			**Community 2 (JI = .210,.205)**^**a**^			**Community 3 (JI = .319,.574)**^**a**^		
	Estimate (SE)	*t*-Ratios	Convergence^b^	Estimate (SE)	*t*-Ratios	Convergence^b^	Estimate (SE)	*t*-Ratios	Convergence^b^
**Network A**									
*Network function of model*									
**Rate constant**									
Period 1	0.992(0.308)			2.378(1.111)			0.612(0.199)		
Period 2	0.681(0.396)			5.093(2.211)			0.603(0.191)		
**Effects**									
Degree	-7.67(0.37)	20.73**	-0.01	-6.08(0.56)	10.86**	-0.01	-9.38(0.37)	24.32**	0.08
Transitive Triads	-3.12(0.57)	5.47**	0.02	-6.30(1.33)	4.74**	0.03	-1.35(0.63)	2.14**	0.03
Degree Assortativity	0.97(0.07)	13.85**	-0.00				1.20(0.11)	10.91**	0.04
Effect on Net of Engagement Ego (egoX)				2.16(0.09)	24.0**	0.05	1.08(0.33)	3.27**	0.06
*Behavior function of model*									
Linear shape	0.974(0.386)			0.974(0.386)			0.974(0.386)		
Quadratic shape	-1.808(0.522)			-1.808(0.522)			-1.808(0.522)		
**Effects**									
Effect on Knowledge of Engagement (effFrom)	1.85(.99)	1.89*	0.08						
**Network B**	**Community 1 (.333,.480)**			**Community 2 (JI = .242,.233)**			**Community 3 (.500,.600)**		
*Network function of model*									
**Rate constant**									
Period 1	3.534(2.337)								
Period 2	0.473(0.320)								
**Effects**									
Density	-3.67(0.84)	4.36**	0.02						
Degree Assortativity	0.27(0.122)	2.21*	-0.01						
Effect on Net of Knowledge Ego + Alt (egoPlusAltX)	0.56(0.28)	1.98*	0.06						
*Behavior function of model*									
Linear shape	1.207(0.518)								
Quadratic shape									

Note. Blank cells indicate effects dropped to obtain significant values for included effects

^a^Jaccard index for Time 1Time 2 and Time 2Time 3

^b^Excellent model convergence is indicated by convergence t-ratios < .1 (Ripley et al., 2014)

^*^p < .05; **p < .01

### Network B

In estimating co-evolution of network structure with knowledge for Community 1 Network B (coalition-committee egos only) the structural effect of transitive triads was dropped as a significant parameter, retaining significant, negative parameters for both degree and degree assortativity. This was the case in Community 2 and 3 Network B with the addition of knowledge having a significant effect on individuals’ likelihood of forming social ties. While Network B for engagement models supported significant effects and had good Jaccard indices, these models were dropped in each community due to the linear combination of engagement scores across time points that limited simulations to extrapolate meaningful effects.

## Discussion

The implementation of WoC childhood obesity prevention interventions requires attention to changes in both social network structure and stakeholder behavior. To date, researchers have placed emphasis on examining social network structure to understand the influence of peer relationships on WoC intervention effectiveness without expanding it to lessons for implementation [19, 21, 23]. While some studies help explore the influence of coalitions’ social network structure on interventions in general, some of which are in childhood obesity prevention, stakeholder behavior must be explored in relation to network structure and in context of implementation at multiple levels.

The exploration of how coalition social network structure and behavior simultaneously change may have implications for WoC intervention implementation. As with other studies, our results indicate that coalition networks become sparser over time. This may, counterintuitively, potentially allow for *better* diffusion of information or behavior among those who stay within the network [33]. This can happen because in very dense networks, redundant information may be more likely to circulate among already-connected individuals than to accommodate novel information from outside. However, there is likely a threshold wherein too sparse a network becomes ineffective. Thus, WoC interventions predicated on mobilizing social networks may consider accounting for this trend by using a network intervention. In this case, network interventions are purposeful efforts to use social networks or social network data to generate and sustain the diffusion of resources or information [34]. In one example, known as the alteration network intervention from Valente’s network intervention typology [35], researchers and community leaders could work together to deliberately “rewire” existing ties among densely connected community groups, increasing cross-sector collaboration and potentially improving the diffusion of information or behavior among those who stay in the network.

Our results indicate that coalition-committee members prefer to associate with those who have more ties. In the absence of a significant behavior effect from either knowledge in or engagement with childhood obesity prevention strategies on network ties, this may point to peer relationships as a stronger driver of changes in knowledge and engagement in our coevolution models. For WoC interventions, this may mean that interventionists should emphasize activities that promote social cohesion and connectedness to support changes in knowledge and engagement. For example, hosting regular convenings that consist of individuals from different sectors as well as local leaders of community groups, that focus on building trust and consensus around goals, may help support the diffusion of knowledge and engagement in childhood obesity prevention.

More granularly, our results indicate similar trends in Network B (coalition-committee members) and Network A (coalition-committee members and first-degree alters) with higher knowledge scores increasing their number of ties over time (Community 2 and 3), but their first-degree alters may play a role in mediating how many of those ties are formed (Community 1). The knowledge subdomains (e.g., perception of one’s ability to personally create changes in the drivers of childhood obesity) may be linked to coalition-committee members’ ability to create and sustain new ties. More research is needed to study this link; however, WoC intervention might benefit from creating components of the intervention that directly address stakeholders’ understanding of the modifiable determinants of childhood obesity, one of the knowledge subdomains.

Engagement with childhood obesity prevention strategies and research did not appear to be significantly influenced by knowledge behavior in context of tie formation. These results support the trend in the models that indicates coalition network structure may be driving changes in knowledge in childhood obesity prevention apart from the engagement domain. WoC interventions with goals to increase enthusiasm for childhood obesity prevention may want to create additional intervention components that directly engage participants more personally to understand their self-assessment of influencing change in their communities, working to improve their sense of agency and motivation to intervene in their organization or community. One route of improving stakeholders’ power and influence is to highlight intervention points within systems maps of childhood obesity that are most proximal to their or their organization’s sphere of influence.

Because coalition-committee demographics vary from community to community (seen in Table 2), WoC interventions may need to be tailored to address baseline and subsequent changes in the coalition network. Departing from Network A, Network B (committee-member only) models indicate more variability in the way individuals show preference for making and sustaining new ties based on knowledge scores, which may be influenced by coalition-committee demographic differences. For example, the higher percentage of individuals who represent the community-based organization sector in Community 2 may play a role in preferential attachment and changes in knowledge scores. Further, while Community 2 and 3’s trend in knowledge scores influencing increases in tie formation may be related to both community’s lower proportion of individuals who identify as non-Hispanic white and smaller land area when compared to Community 1, results indicate that coalition-committee characteristics (e.g., baseline density) were more relevant in determining model variability. When implementing WoC interventions, this may indicate a need to focus on the unique social characteristics of key stakeholders participating in the intervention. Finally, each coalition had a different focus that may have increased model variability; however, this link is not well-studied. For example, future research could compare coalitions that focus on the evaluation of early care programs and coalitions that focus on increasing youth physical activity, as well as their approaches or activities in understanding these areas, to better contextualize how knowledge, engagement, and social networks coevolve. Future research on the link between knowledge, engagement, and social networks would benefit from also collecting a rich history of community culture that can contextualize simulation results.

Variability in the models may point to several implications for implementing WoC childhood obesity prevention interventions. First, childhood obesity prevention interventions that utilize diffusion [25, 33] as a part or primary piece of the implementation theory would benefit from iteratively tailoring the implementation of the intervention. From this perspective, tailoring could consist of identifying coalition structure and behavior variability and intentionally pairing stakeholders together to create potential for making new connections and sharing information and resources [35]. Second, despite model variability, the trends across communities indicate potential for stochastic actor-oriented models to be used as a tool for iterative, rapid-response simulation to improve the implementation of WoC childhood obesity prevention interventions. For example, researchers could develop stochastic actor-oriented models after two or more waves of data collection, examine the general coevolution of coalition structure and behavior, and refine the implementation of the multi-level intervention accordingly. Examples of what refinement may look like can be found in articles that discuss network interventions [34–36].

More longitudinal research is needed to explore additional coevolution interactions that impact WoC intervention implementation and coalition mobilization around childhood obesity prevention. While researchers have focused more heavily on coalitions’ social network structure in their model development, including additional behavioral variables (e.g., intrapersonal, psycho-social constructs) could uncover key coalition social dynamics that can be harnessed to improve the implementation of WoC childhood obesity prevention interventions. Finally, future efforts to build coevolution models, or simulation models more generally, should attempt to further examine whether simulation can be used to (1) tailor interventions to specific community contexts; and/or (2) contribute to the development of network interventions.

### Limitations

First, while this study utilizes several cases to examine coevolution in coalitions, its findings are not necessarily generalizable. Instead, these findings can inform theory related to coalition efficacy, improving the use of research evidence, and obesity prevention. Second, due to the nature of coalitions as rapidly fluctuating groups embedded in shifting community landscapes, it is possible that events other than coalition and intervention activities were responsible for changes seen in each model. Thirdly, related to this second point, sample size for Network A varied considerably at each time point and decreased overall. However, the Jaccard Index, which is used to assess sample variability between waves for simulation models, did not indicate that this variability was an issue. Finally, while only three time points were used to estimate simulation models using the RSiena package in R, additional time points would provide more confidence in the results. Following standard practice, goodness of fit tests were applied to ensure simulations fit observed data [31].

## Conclusion

The implementation of WoC obesity prevention interventions can be further tailored by iteratively using simulation models that provide potentially vital prospective information on coalition social network structure and behavior. Coevolution models could also aid coalitions in understanding how member participation in coalition activities supports their overall progress toward improving coalition implementation of childhood obesity prevention interventions. Our coevolution models from three communities indicated that coalition-committee members and coalition-committee members plus their first-degree alters are more influenced by social network structure than knowledge in and engagement with childhood obesity prevention. Our models also indicated that individuals with higher levels of knowledge in childhood obesity prevention in each network showed preference for making and sustaining new ties, to varying degrees. That is, the preference to be connected to others with more ties is mediated by their alters’ level of knowledge in childhood obesity prevention. The overall variability in the models indicates that the implementation of childhood obesity prevention interventions must be tailored to their specific community context and that simulation may be used as a tool to understand how implementation needs to be tailored. 

## Data Availability

The datasets used and/or analyzed during the current study are available from the corresponding author on reasonable request.

## Abbreviations

RSiena: Simulation Investigation for Empirical Network Analysis
SDCD: Stakeholder-Driven Community Diffusion
SNA: Social Network Analysis
SNS: Social Network Simulation
WoC: Whole-of-Community

## Footnotes

1 In RSiena, this assumption is defined by selecting the function “modelType = 3” for non-directed networks, also known as the unilateral initiative and reciprocal confirmation model specification. This model type views tie formation as one actor taking initiative and proposing a new tie or dissolving an existing tie; if the actor proposes a new tie, the other must confirm for the tie to be created. For tie dissolution, confirmation is not required (see RSiena Manual, page 54 for more information on model type).

2 These functions operate by comparing the observed values at the ends of the measured periods with the simulated values for the ends of the periods. The differences in simulated and measured values at the end of each period are assessed by using the Mahalanobis distance to combine the auxiliary statistics.

3 egoX, altX, effFrom, and related effect names are derived from the RSiena Manual [30] and describe different influence effects on or between network structure and behavior. Definitions of these terms can be found starting on page 123 in the manual.
